# Functional Implications of Local DNA Structures in Regulatory Motifs

**DOI:** 10.1155/2013/965752

**Published:** 2013-05-14

**Authors:** Qian Xiang

**Affiliations:** School of Information Science and Technology, Sun Yat-Sen University, Guangzhou 510275, China

## Abstract

The three-dimensional structure of DNA has been proposed to be a major determinant for functional transcription factors (TFs) and DNA interaction. Here, we use hydroxyl radical cleavage pattern as a measure of local DNA structure. We compared the conservation between DNA sequence and structure in terms of information content and attempted to assess the functional implications of DNA structures in regulatory motifs. We used statistical methods to evaluate the structural divergence of substituting a single position within a binding site and applied them to a collection of putative regulatory motifs. The following are our major observations: (i) we observed more information in structural alignment than in the corresponding sequence alignment for most of the transcriptional factors; (ii) for each TF, majority of positions have more information in the structural alignment as compared to the sequence alignment; (iii) we further defined a DNA structural divergence score (SD score) for each wild-type and mutant pair that is distinguished by single-base mutation. The SD score for benign mutations is significantly lower than that of switch mutations. This indicates structural conservation is also important for TFBS to be functional and DNA structures will provide previously unappreciated information for TF to realize the binding specificity.

## 1. Introduction

Gene expression is regulated mainly through specific interaction of transcription factors (TFs) with gene promoter elements. Although a large amount of various TF binding sites (TFBS) have been characterized through targeted low-throughput experiments or high throughput methods, such as chromatin immunoprecipitation coupled to sequencing (ChIP-seq) and protein binding microarray (PBM) assays [1, 2], there is no distinct nucleotide sequence which is shared by all recognized TFBS and most TFs may interact with many diverse sequences. The specificity of TF bindings is commonly represented by position weight matrices (PWMs), the components of which give the probabilities of finding each nucleotide at each binding site position [3].

While nucleotide sequence might be the key determinant for functional TF-DNA interaction, local DNA structure is also important as shape readout is one of the main recognition modes that are used by a large class of TFs when they scan the genome for regulatory interaction [4]. Although the DNA structure is somehow dependent on nucleotide sequence, similar sequence does not guarantee similar structure and vice versa. Divergent DNA sequences can share a similar local structure. Conversely, similar DNA sequences can adopt distinct local structures [5–7]. There is strong evidence that TFBS with different nucleotide order can also be recognized by the same TF and many TFs are capable to interact with diverse types of DNA sequences [1]. These observations indicate that although these TFBS are different on nucleotides sequence, they may be similar in structure and perform similar biological functions.

Here we ask whether there are some particular local structures, which are associated with the binding site of TFs. To address this question, we use hydroxyl radical cleavage pattern as proxy for the local structure of each TFBS [5]. We show here that there are DNA structural elements that are highly enriched in TFBS and these structural elements are not predictable on the basis of DNA sequence information alone. Our results suggest that DNA structures will provide previously unappreciated information for TF to realize the binding specificity and consideration of local DNA structure as well as nucleotide sequence will be important to understanding the regulatory interaction of TF-DNA.

## 2. Materials and Methods 

### 2.1. Data Sets Used for This Work

Yeast genome sequences are downloaded from the website of http://www.yeastgenome.org/. The PWMs for 114 yeast TFs are derived from in vitro experiments in the literature [8], which denote the inherent sequence affinities of TF. The golden-standard set for true TFBS sites are achieved from file p005_c3.gff downloaded from [9]. Only 89 TFs with at least 10 annotated TF binding sites will be analyzed further.

### 2.2. DNA Structural Profile and Divergence Score (SD Score)

Hydroxyl radical is a nearly ideal chemical probe for mapping genomic DNA structure. Here, all possible single-base substitutions for each putative TFBS were generated. We use the proposed algorithm in [5, 6] to predict the hydroxyl radical cleavage pattern for each wild type and mutant, respectively. The cleavage pattern for each sequence provides a measure of local DNA structure and is regarded as the DNA structural profile. The pairwise Euclidean distance for putative TFBS *i* between the DNA structural profiles for the wild-type *W*_*i*_ = [*w*_*i*1_,…*w*_*iN*_] and mutant *M*_*i*_ = [*m*_*i*1_,…*m*_*iN*_] pair is defined as

(1) dE(i)=∑j=1N(wij−mij)2,

where *N* is the length of TFBS. For the convenience of the comparisons among different TFBS, Euclidean distances are divided by their motif lengths, respectively:

(2) SD-score=1N∑j=1N(wij−mij)2.

Therefore the DNA structural divergence score (SD score) is defined as the normalized Euclidean distance.

### 2.3. Calculation of DNA Structure and Sequence Conservation

We measure the conservation in DNA structure and sequence in terms of information content, which is defined in the same way as in [6, 10]:

(3) R(l)=Emax⁡−H(l)Emax⁡,

where *E*_max⁡_ is the maximum amount of uncertainty possible at any given position (in bits), and *H*(*l*) is the uncertainty at position *l* based on the observed binding sites. The decrease in uncertainty represents the total information content at the position after the binding site alignment [10]. In order to compare the conservation between sequence and structure directly, information was divided by their maximum possible entropies, respectively, so that *R*(*l*) represents the amount of normalized information content present at position *l* [6].

### 2.4. Enrichment Analysis

All of the wild-type and mutant pairs are classified into three datasets denoted as benign mutations, switch mutations, and loss mutations. We adopted the same definition of three scenarios in [11] to classify single-base mutations in TF binding sites: (i) Benign mutation—the mutant sequence is also recognized by the same TF and the substitution is expected to have a very mild effect on DNA structure. (ii) Switch mutation—the mutant sequence is no longer recognized by the original TF, but it is recognized by an alternative TF. (iii) Loss—the mutant binding site is no longer recognized by any TF. 

Enrichment of lower SD score in benign mutation pairs and higher SD score in switch mutation pairs were evaluated in a simple and elegant way introduced by [12]. First, all pairs with possible single-base substitutions to the putative TFBS were exhaustively enumerated and serve as background control. The DNA structural profiles were calculated and compared for each pair. Next, the Euclidean distance between pairwise structural profiles was computed and defines the DNA structural divergence scores, which are divided into different bins. The fraction of benign or switch mutation pairs in each bin was calculated as *T*. The fraction of background control pairs in each bin was also calculated as *B*. The enrichment score is then defined as

(4) E_score=TB.

By this scheme, no relation between mutation types and SD score will have an enrichment of 1. Correlation between mutation types and SD score will have value ≫1, while those mutation types that show significant anticorrelation with respect to SD score will have a value ≪1. In order to determine the distribution of the enrichment score under the null hypothesis of no enrichment, the same amount of pairs as those of benign mutations or switch mutations are randomly sampled from the background dataset by 1,000 times and significance *P* value was evaluated by hypergeometric tail probability. 

## 3. Results and Discussion

### 3.1. Many DNA Structural Patterns in Motif Having Less Sequence Similarity

As a first step towards establishing a general scheme to find hydroxyl radical cleavage patterns that are shared by a set of binding sites of transcription factors, we examined the DNA consensus structural pattern of the yeast Asparagine-rich Zinc-Finger factor AZF1 as an example, which is the glucose-dependent positive regulator of CLN3 transcription in *S. cerevisiae* genome [13]. TF binding sites with different sequence composition can have similar cleavage patterns. For example, Greenbaum et al. showed that common structural motifs were detected in a large collection of DHSs that are found in the ENCODE regions of human genome [6]. Inspired by this work, we here ask whether similar structural motif exists for TF binding sites. Using the same methodology as mentioned in [6], the range of predicted continuous-value hydroxyl radical cleavage intensity is divided into 50 bins. The probabilities of finding each discretized intensity level at each binding site position are calculated and represented as a position frequency matrix. 

**Figure 1:**
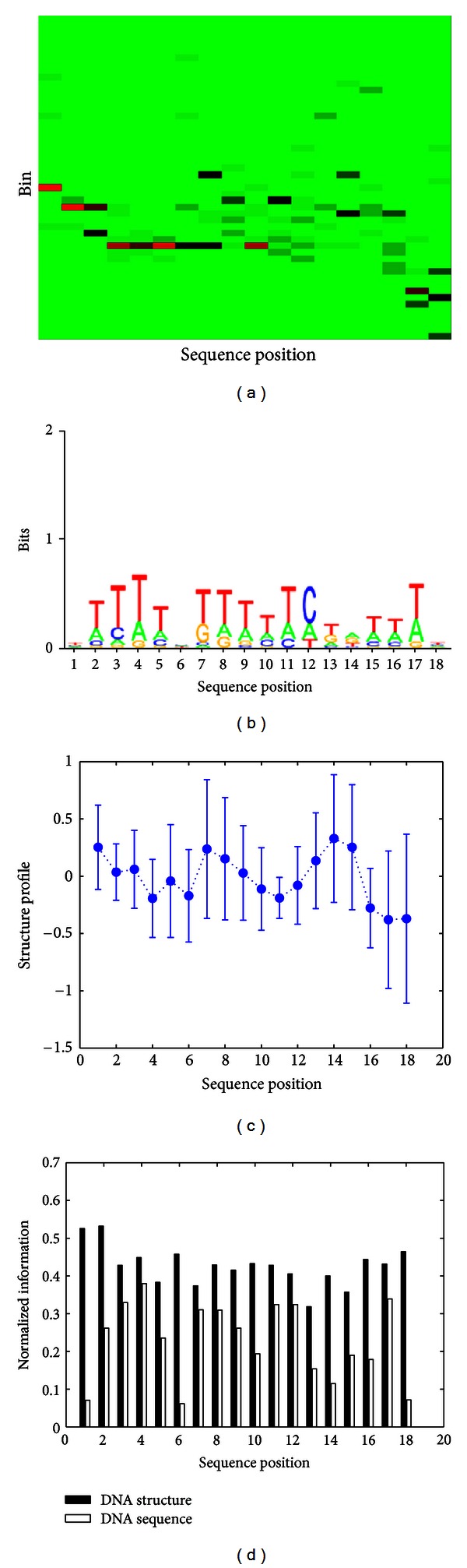
Analysis of the representative AZF1 consensus structural pattern. (a) Heat map of AZF1 consensus structural pattern found in putative TFBS. (b) Sequence logos of AZF1 found in putative TFBS. (c) Mean and standard deviation of predicted hydroxyl radical cleavage patterns. (d) Conservation of structure versus sequence in AZF1.

The representative structural motif is depicted as heat map in Figure 1(a). Here, *x*-axis represents sequence position in the TFBS and *y*-axis represents cleavage value bins. Green cells in the heat map indicate that no cleavage values for bin *Y* at position *X* are present, whereas red cells indicate a large proportion of the cleavage values in that bin. Obviously, each column would be uniformly colored if cleavage values were randomly distributed. For comparison, the corresponding sequence alignment among all the TF binding sites is also examined and shown in Figure 1(b). We found little similarity between nucleotide patterns and structural patterns. 

To further investigate the above point, we plotted the mean predicted hydroxyl radical cleavage pattern values along with their corresponding standard deviation for each position of AZF1 in Figure 1(c). The mean cleavage intensity at any given position closely mirrors what is indicated in the heat map. Moreover, we also calculated the similarity for both sequence and structure of AZF1 in terms of information content. More specifically, we calculate the maximum entropy minus the observed entropy at each position of the alignment, normalized by the maximum entropy. Entropy is a measure of degeneracy or uncertainty. Information is a measure of the decrease of uncertainty. Therefore, an alignment with higher information content is more conserved [6]. According to the result of AZF1 shown in Figure 1(d), it is obvious that the structural alignment contained more information than the corresponding sequence alignment in all positions. 

Encouraged by our ability to predict the consensus structural pattern within TF binding sites, we attempted to generalize these procedures in order to find universal DNA structural properties for various transcriptional factors. Towards this end, we compiled a collection of 5587 putative TF binding sites which are likely to be functional TFBS in *S. cerevisiae* genome [9]. We next exploited the motifs dataset in order to generate the DNA consensus structural patterns and compare the conservation between sequence and structure in terms of information content. We calculated the total information content of the cleavage pattern alignment for each TF along with the corresponding information content of the nucleotide sequence alignment. 

**Figure 2:**
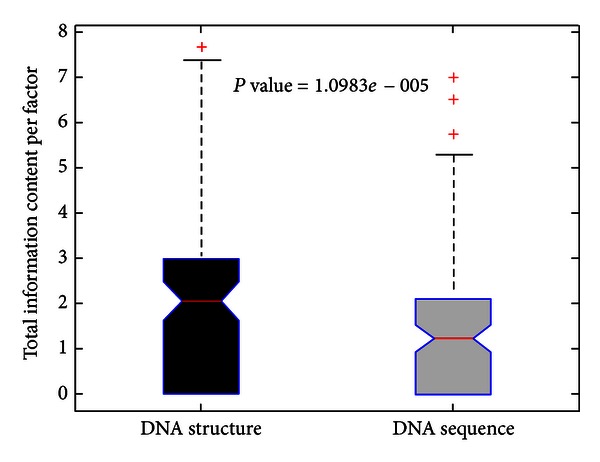
Total information content per factor in structure versus sequence.

As indicated in Figure 2, the structural alignment contained significantly more information than the corresponding nucleotide sequence alignment for most of the transcriptional factors (*P*-value < 1.1 × 10^−5^, two-sample Kolmogorov-Smirnov test). It suggests that DNA structures are more conserved than DNA sequences for most of the TFBS.

**Figure 3:**
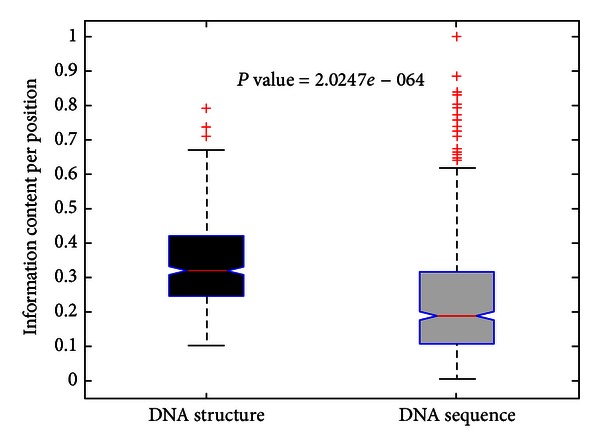
Information content per position in structure versus sequence.

 Furthermore, we compared the conservation between sequence and structure by the information content per position. The results in Figure 3 show that majority of positions have more information in the structural alignment as compared to the sequence alignment (*P*-value < 2.1 × 10^−64^, two-sample Kolmogorov-Smirnov test). The higher information content observed in the structure compared with that in the sequence is suggestive that conservation in sequence can only partly explain conservation in the DNA structure. Most of the additional conservation can be attributed to structural functional regulatory of TF binding sites.

### 3.2. Effect of Binding Site Substitution on DNA Structure

We next want to quantify the effect of binding site variation on DNA structure. For each of the collected 5587 putative TF binding sites, we exhaustively enumerated all possible single-base substitutions and measured the similarity between profiles for all wild-type and mutant sequence pairs that differ only by a single substitution. 

Similarly as those three scenarios which were defined in [11], we can distinguish all pairs of sequences which differ by a single substitution into three types. Among all of the 133885 unique wild-type and mutant pairs which are generated by single-base substitutions for all of the collected 5587 putative TFBS, the substitutions in 4959 pairs, which do not change the original TF-DNA interaction relationship, are regarded as benign mutations. On the other hand, there are 1706 pairs whose original motif and the substituted sequence are both members of the TFBS dataset, but they are recognized by different TFs. We consider these mutations as switch mutations. All the other 127220 pairs are loss mutations where the substitutions result in a sequence that is no longer in the collected TFBS dataset. Overall the analysis revealed that it is apparent that the majority (95%) of single nucleotide substitutions result in loss of functional binding site.

Here we define DNA structural divergence score (SD score) for each mutation as the normalized Euclidean distance between two structural profiles. Extreme DNA structural divergence can be attributed to several possible factors, such as DNA sequence substitution, chromosomal structure, or positive selection for DNA local structure. Thus we sought to determine the potential sources accounting for the observed divergence between DNA structures for single-base substitution pairs. Parker et al. have showed that single-base substitutions have a wide range of effect to DNA structure from minor to drastic [7]. Inspired by their work, we here ask whether there is an enrichment of small SD score that exists for benign mutations compared to the background distribution. 

**Figure 4:**
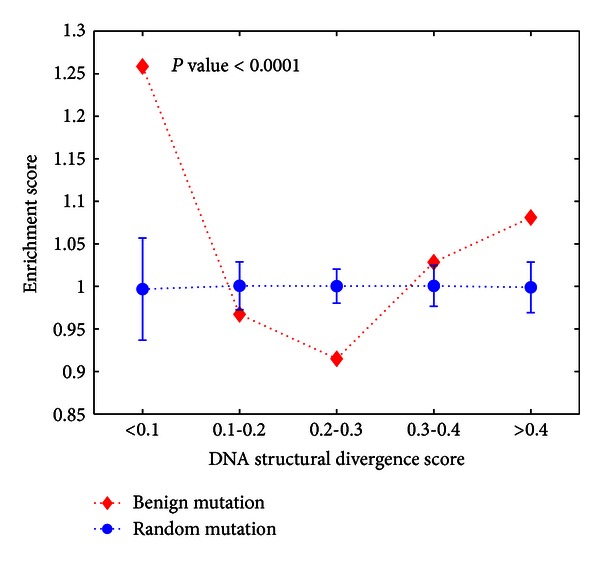
Benign mutations show extremly low DNA structural divergence.

We then collected a total of 133885 pairs with single-base substitutions to serve as a background control. We divided DNA SD score into different bins and computed the enrichment scores for benign mutations in each bin. The enrichment score is the fraction of benign mutations in each bin, divided by the fraction of all mutations in the same bin. To determine the distribution of the enrichment score under the null hypothesis of no enrichment, we generated 1,000 data sets with 4,959 randomly sampled mutations.  Figure 4 clearly shows that loci with extremely small DNA SD score (<0.1) were significantly enriched for benign mutations (*P*-value < 0.0001, hypergeometric tail probability). 

**Figure 5:**
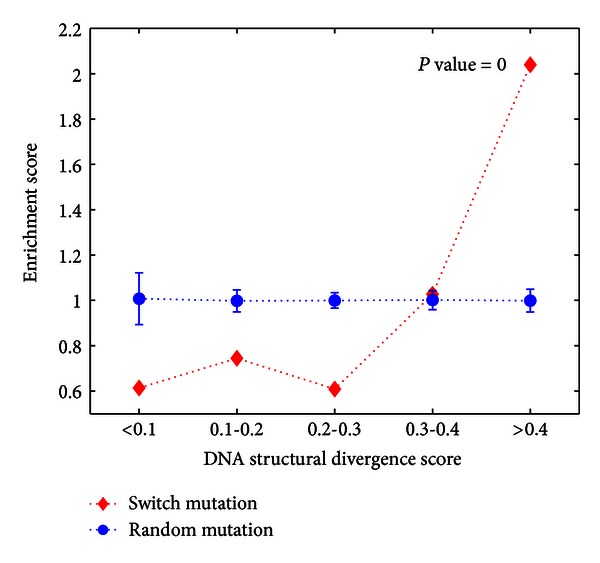
Switch mutations show extremely large DNA structural divergence.

Similarly, we also studied the situation for switch mutations by generating another 1,000 datasets with 1706 randomly sampled mutations. On the contrary, the results in Figure 5 indicated that loci with extremely large DNA SD score (>0.4) were significantly enriched for switch mutations (*P*-value *≈* 0, hypergeometric tail probability). All these findings clearly indicate that DNA structural conservation is important for TFBS to be functional and the DNA structural patterns change a lot between different TFs.

## 4. Conclusions

The binding nature of transcription factor to specific locations in the genome is one of the most important features for gene regulation in cells but remains poorly understood. We here showed that the predicted hydroxyl radical cleavage pattern can be successfully used to provide putative DNA structural profiles for each TFBS. The comparison results clearly demonstrated that higher information content at the structure level was observed than that at the sequence level for most of the TFs and in the majority of positions. Moreover, we compared the DNA structural profiles between wild-type and mutant motifs and assessed how drastically each type of substitution affected DNA structures. The statistically analysis indicated that not all effects of mutation are equal: for example, benign mutations are less likely to change the DNA structures, compared to switch mutations. We therefore speculate that some of the functional information in the TFBS is conferred by DNA structure as well as nucleotide sequence. One future implication of these findings is that it may point the way to improved accuracy in the prediction of the functional regulatory interactions. Our results may also provide aid to distinguish which mutation in promoter elements is more likely to cause abnormal transcription by affecting the DNA structure.
